# Fixing the earth: whole-systems thinking in Silicon Valley’s environmental ideology

**DOI:** 10.1080/24701475.2024.2416295

**Published:** 2024-10-17

**Authors:** Rianne Riemens

**Affiliations:** Radboud Institute for Culture & History, Faculty of Arts, Radboud University, Nijmegen, The Netherlands

**Keywords:** Silicon Valley, climate crisis, ideology, ecomodernism, whole-systems thinking, techno-optimistic discourse

## Abstract

Today, American tech actors express optimistic ideas about how to fix the Earth and halt climate change. Such “green” initiatives have in common that they capture the world in systems and propose large systemic, and mostly technological, solutions. Because of their reliance on techno-fixes, representatives of Silicon Valley express an ideology of ecomodernism, which believes that human progress can be “decoupled” from environmental decline. In this article, I show how “whole-systems thinking” has become a key discursive element in today’s ecomodernist discourses. This discourse has developed from the 1960s onwards – inspired by cybernetic, ecological and computational theories – within the tech culture of California. This paper discusses three key periods in this development, highlighting key publications: the *Whole Earth Catalog* of the 1960s, the *Limits to Growth* report in 1972 and the cyberspace manifestoes of the mid 1990s. These periods are key to understand how techno-fixes became a popular answer to the climate crisis, eventually leading to a vision of the world as an ecosystem that can be easily controlled and manipulated, and of technological innovation as harmless and beneficial. I argue that “whole-systems” thinking offers a naive and misleading narrative about the development of the climate crisis, that offers a hopeful yet unrealistic perspective for a future threatened by climate change, built on a misconception of Earth as a datafied planet.

## Introduction

In “The Techno-Optimist Manifesto” (2023) venture capitalist Marc Andreessen argues why we should all be techno-optimists, especially if we are worried about the future impact of the climate crisis. According to Andreessen, promoting unlimited technological progress is the only option: “there is no inherent conflict between the techno-capital machine and the natural environment”. If we generate unlimited clean energy, we can improve the natural environment, whereas a “technologically stagnant society ruins it” (Andreessen, 2023). This is possible, he writes, because technologies enable processes of dematerialization and will eventually lead to material abundance. And, “We believe the market economy is a discovery machine, a form of intelligence—an exploratory, evolutionary, adaptive system” (Andreessen, 2023). The manifesto thus conceptualizes technology as immaterial and the capitalist economy as an evolutionary system: it presents techno-fixes as a harmless form of environmental action, and economic growth as an inevitable process that political powers should not interfere with.

The “Techno-Optimist Manifesto” is an example of a form of techno-optimism that places full trust in the potential of capitalist tech companies to help humanity “innovate” its way out of a climate crisis. Andreessen (2023) cites historical figures including Buckminster Fuller, Stewart Brand, Douglas Engelbart and Kevin Kelly as the inspiration for his manifesto, showing that the work of these figures and their communities is being remixed and reappropriated into the future visions of contemporary techno-optimists. In this article, I analyse how the belief in the environmental potential of techno-fixes is engrained in the ideology and history of “Silicon Valley” and is discursively constructed through a language of “whole-systems thinking”. I use the concept of whole-systems thinking as a lens to study how simplified notions taken from whole-systems theory and cybernetics played and still play a key role in techno-environmental discourse in the post-war era in the United States. I zoom in on three key events that help explain the origins and evolution of popular whole-systems thinking: the *Whole Earth Catalog* community led by Stewart Brand in the 1960s, the *Limits to Growth* report by the Club of Rome in the 1970s and the cyberlibertarian community in the 1990s. I will show how a new language emerged that used simplified notions of systems-thinking to promote the idea that technology would help understand, manage and save a planet in peril.

Through a discourse analysis of primary sources and literature review I present a critical reading of these events in the light of today’s techno-optimistic environmental discourse. My corpus exists of a number of primary sources, including the aforementioned “Techno-Optimist Manifesto” (2023), *Limits to Growth* report (Meadows et al., 1972), editions of the *Whole Earth Catalog* and *CoEvolution Quarterly*, Barlow’s *Declaration of the Independence of Cyberspace (1996)*, texts by Kevin Kelly (1998) and Stewart Brand (2009) and An *Ecomodernist Manifesto* (Asafu-Adjaye et al., 2015). I have discursively analysed these sources for their discussion of systems thinking as well as environmental concerns. By analysing how whole-systems thinking became a popular way of addressing environmental issues, I aim to provide a “post-war genealogy” (Pedwell 2022) of the term and critique today’s promises about how tech can save the climate. As Johnston (2020) has argued, tracing the development of a cultural perception of trust in techno-fixes reveals a complex and multi-sided history. I claim that the environmental dimension of techno-optimistic discourses requires a critical reconsideration of the ideological underpinnings of Silicon Valley, described as the “Californian Ideology” by Barbrook and Cameron (1996). I will demonstrate how ecomodernism, including its belief that human progress can be “decoupled” from environmental decline, allows us to better understand, and critique, the environmental ideology of Silicon Valley.

I will first expand on contemporary ecomodernism and present my thesis that “decoupling” nature from culture has come to underlie whole-systems thinking in contemporary techno-optimistic discourse. In the following three sections, I highlight a few historical moments to demonstrate the development of the cultural perception of techno-fixes, specifically as a means of managing the environment. I show how whole-systems thinking became popularized by the Whole Earth community, got incorporated in environmental debates through the *Limits to Growth* report and is reflected in cyberutopian dreams about immaterial societies. Building on my necessarily brief history, I argue that techno-fixes can be strategically presented as ideal solutions if the world and environment are imagined as simple systems and technology as immaterial and harmless. Finally, I return to contemporary US tech culture and argue that it is shaped by, and co-shapes, the ideology of ecomodernism in which nature and culture are decoupled. I conclude that this worldview expresses itself today in corporate visions, resulting in a false hope about how to innovate our way out of the climate crisis.

## Ecomodernism and the strategy of “decoupling” nature from culture

While Andreessen’s manifesto may be radical in its optimism and its adaptation of systems-thinking, his rhetoric in times of climate crisis is not unique in Silicon Valley. Building on existing research, I understand Silicon Valley as both a Californian region and a broader American cultural phenomenon (Kenney, 2000; O’Mara, 2019; Streeter, 2011; Turner, 2006). Another representative of this culture is Amazon founder Jeff Bezos, who employs similar forms of techno-optimism and systems-thinking to promote his Bezos Earth Fund. On the website, the Fund describes “a systems approach”, introducing its Systems Change Lab that “helps us monitor the necessary shifts, learn about ingredients for transformation, and catalyze change with our partners and coalitions vying for a new way forward” (Bezos Earth Fund, 2023). The Fund proposes a technological solution to accomplish what it calls a necessary shift and transformation to solve the climate crisis in the 2020s. Another example of systems thinking is Microsoft that uses its cloud platform Azure to host “The Planetary Computer”: a database allowing researchers to “monitor, model and manage” ecosystems (Microsoft (Producer), 2020).

Techno-optimism and systems-thinking are part and parcel of Silicon Valley. Tech companies and leading figures of Silicon Valley recognize environmental decline and humanity’s dependence on natural ecosystems, but discursively place their own companies and production processes outside of this system. I call this strategy “decoupling”: the discursive process through which Silicon Valley actors emphasize certain material connections while erasing others. Originally, the term decoupling is used in the ecomodernist movement, which has risen as a school of thought in the beginning of the twenty first century, exemplified by the foundation of the Californian Breakthrough Institute by Ted Nordhaus and Michael Shellenberger (*About Us*, 2023). Ecomodernists believe that human progress can be “decoupled” from environmental decline, because, as they write in their manifesto, there are no “fixed physical boundaries to human consumption” if only we use “proper management” (Asafu-Adjaye et al., 2015, p. 10). Humanity can “achieve peak human impact without intruding much further on relatively untouched areas. Nature unused is nature spared” (p. 19). Several authors have pointed out that this discursive separation of culture from nature is dangerously reductive and overlooks the detrimental consequences our current economies have on natural ecosystems (Crist, 2016; Hogan, 2018; Kallis & Bliss, 2019). I argue that decoupling—both as a discursive strategy and as an ecomodernist belief—is a crucial part of techno-optimist discourse and whole-systems thinking that portrays the world as an ecosystem that can be easily controlled and manipulated. In the following three sections, I demonstrate how different elements of this contemporary narrative and ideology are rooted in the history of American tech and environmental discourse.

## The 1960s: from the *Whole Earth* to Whole systems

In the 1960s, the San Francisco Bay area became both the centre of the American counterculture, drawing people from all over the US to the area, as well as the centre of computer research, taking place at local research labs and universities. This region later became known as Silicon Valley, a term first used in 1971 to describe the concentration of tech industry in the area (O’Mara, 2019). Although it was not the only hub for either of these movements, this area was unique in the way that potentially oppositional communities such as the counterculture and research schools interacted and shaped one another, leading to a new movement of tech-savvy community-oriented individuals called “new communalists” (Turner, 2006). It is in this particular convergence that the language of systems-thinking emerged in popular writing about technology, culture, and ecology.

The new communalists were inspired by developments in the military-industrial complex, but also by research fields such as cybernetics and ecology. For them, the non-military application of technologies and the language of system-thinking provided stability and hope against the backdrop of WWII and the Cold War. Fred Turner (2006) has convincingly argued that there was a lot of interaction between the counterculture and military-industrial complex, despite their seemingly conflicting values and interests. Organizations such as Stewart Brand’s *Whole Earth Catalog* (1968-1972), Douglas Engelbart’s research group at Stanford University, and the computer hobbyists of the Homebrew Computer Club all met frequently and exchanged ideas (Turner, 2006, p. 106). Bridging the gap between environmentalists and tech researchers, Stewart Brand created a “Whole Earth” community, in which an influential story emerged about the potential of technology for human freedom and harmony, as well as control over life, planet Earth, and even outer space (Kirk, 2007; Turner, 2006). Such techno-optimism proliferated throughout this decade and remained a central element of Silicon Valley ever since.

The discourse and ideas that circulated within this community were heavenly influenced by cybernetics: the research field that had emerged in the 1940s and specialized in systems thinking and is often seen as the birth ground of the information age (Hayles, 1999; Kline, 2015). Scientists such as Norbert Wiener, John von Neumann and Margaret Mead, with backgrounds in fields such as biology, anthropology and communication science, developed a “theory of information and control applying equally to animals, humans, and machines” (Hayles, 1999, p. 7). Homeostasis, which traditionally addressed the ability of beings to maintain a steady state, was understood as a concept that could help build and understand machines, with the feedback loop theorized as a flow of information feeding back into a system (Hayles, 1999, p. 8). Cybernetic insights, especially the idea of substituting human control by technologies, led to many reconsiderations of how systems worked and could be managed (Johnston, 2020, p. 54). The impact of cybernetics on twentieth century ideas about subjectivity, machines, humans and nonhumans was immense. It led to a fundamentally new perspective on human beings as information-processing entities, much like machines (Hayles, 1999). Thomas Streeter describes it as such:

The idea that thinking was like information processing laid the foundation for both the idea that computers could become minds, the cornerstone of the field of artificial intelligence, but also, reciprocally, that people could be understood and organized into centrally controlled communication systems, the cornerstone of the field of systems science. (Streeter, 2011, p. 31)

This vision included a strong appreciation of the capabilities of computers and their pote`ntial for the management of not only military machines, but also humans, and even nature. The idea to think of computers as minds and of the world as a system—in other words, to think in terms of patterns of information—was revolutionary. Some saw it as an opportunity for a more efficient and intelligent work sphere made possible by technology, while others saw the potential for a harmonious, ecological society (Turner, 2006). In both cases, the cybernetic vision of the world offered a sense of control, safety, and optimism about the future.

While rapid technological developments induced optimism from the early twentieth century onwards, in the second half of this century seductive and accessible tales of technological progressivism disseminated among a wide American audience (Johnston, 2020). Although the post-war period was also characterized by fear and uncertainty, the social value of technological innovation became widely accepted (Johnston, 2020, p. 50). Such tales were constructed along specific rhetorical lines, equating technology and innovation with progress and imagining engineers and technicians as the managers of a soon-to-be transformed society (Johnston, 2020, pp. 13–14). In the context of the military-industrial complex, Edwards (1996) argues that a worldview emerged in which two types of discourse came together: a “closed-world” and a “cyborg” discourse. Whereas the first speaks of military strategies in terms of “centralized command and control”, the latter articulates “metaphors of minds as computers” (p. xiii). Edwards describes how not only a specific situation of warfare, but eventually the entire world of global politics came to be seen as a closed system, in which computers would allow for centrally controlled power (pp. 7–8). Forms of systems-thinking and techno-optimism thus became part of mainstream discourse, using simplifications of the systems-theories invented and employed by cyberneticians.

The new “Whole Earth” movement thus arose from two rather different communities: people from the military focused on the development of new technological systems as methods of warfare and the free-spirited, anti-establishment hippies focused on the exploration of the self. Merging together they combined in a counter-culturalist movement that saw computers as agents of cultural change (Streeter, 2011; Turner, 2006). The counter-culturalists of the Whole Earth community were key in the popularization of techno-optimism and systems-thinking. Their ideas about technologies as creative tools were crucial for the wider acceptance of technology as tools for everyday life. Yet, the notion that technology would help to “control” the world remained popular. Brand famously opened every edition of the *Whole Earth Catalog* with the words “we are as gods and might as well get used to it” and expressed the wish for each individual to “shape his own environment” (Brand, 1969).

The Whole Earth community used the language of systems thinking to present the possibilities of technology to a wider audience. Every *Catalog* had a “Whole Systems” section, inspired by the ideas of designer Buckminster Fuller and others (Kirk, 2007, pp. 56–57). The focus on whole-systems correlated with the community’s interest in environmental themes, for example reflected in the promotion of renewable energy, energy conservation and energy-efficient houses and products (Kirk, 2007, p. 214). In fact, historian Andrew Kirk summarizes their visions as a “whole-systems worldview” (p. 64). Emphasizing the influence of cybernetics on the environmental philosophy of the *Whole Earth Catalog*, Kirk (2007, p. 63) argues that the Whole Earth community distanced itself over time from the hippie “wilderness-based movement” and instead propagated an optimistic, pragmatic environmental philosophy that linked “science, technology, ecology, design, and postscarcity”.^1^ In this worldview, the world was understood as “a system of invisible forces” with which humans should try to live in harmony with, so that the Earth as a system could achieve a state of stability (Turner, 2006, p. 83).

Such an environmental approach was emblematic for the changes and debates in the field of ecology, influenced by the work of cyberneticians. In the 1960s, ecologists started using the term “ecosystem ecology” to replace the romanticized image of ecologists as “glorified bird watchers” (Kingsland, 2005, p. 179). Ecosystem ecology “welcomed applications of new techniques in applied mathematics, looked to physics to provide basic principles for ecology, and pushed ecology into the emerging computer age with enthusiasm” (p. 179). The abstract concept of the ecosystem made it applicable not only to natural environments, but also to human communities (Kingsland, 2005, pp. 191–192).

This form of ecosystem ecology resonated with the Whole Earth community. Stewart Brand and other communalists were optimistic about the potential of appropriate technological applications. This attitude was quite different from writers such as Lewis Mumford, Theodor Roszak and Jacques Ellul, who were critical of the ongoing processes of rationalization and centralization and the technocratic worldview of the social world as an automated machine (Turner, 2006, pp. 28–29). By developing a whole-systems language inspired by the computer industry, cybernetics and ecology, this Whole Earth discourse supplied a specific vernacular to speak of technology and nature in similar terms. Building on Kirk’s (2007) notion of the whole-systems worldview, I argue that this discursive change is key to understand the rise of techno-optimistic discourse. While Kirk rightfully credits the Whole Earth community for the creation of a new environmentalism that welcomed pragmatic technological solutions, my analysis shows that it also paved the way for later techno-optimistic discourse. In a sense, this discourse *naturalized* technology: not only human-tech relations came to be celebrated, but a positive connection was drawn between the domains of technology and nature, emphasizing the ecological potential and even the “naturalness” of technological solutions. In other words: they became discursively “coupled”. Such a conceptual appropriation of concepts such as “nature” and “ecosystem” has become a common way to promote new technologies as environmentally friendly (Hopster et al., 2024). Whole-systems thinking thus provides an early example of how people within the Californian tech culture came to understand technology as a force of nature, instead of as the opposite of nature. The emerging discourse shaped the community’s discussion about self-exploration and human-world relations, acknowledging relations of dependency between human and nature while still describing humans as masters over the environment.

## The 1970s: introducing world models to a degrowth agenda

The late 1960s and early 1970s mark the rise of the environmental movement in the United States, with the first Earth Day in April 1970 as a defining moment, when twenty million Americans protested against environmental destruction (Yeo, 2023). Under the influence of scientific and popular publications during the 1960s and 1970s, such as Rachel Carson’s *Silent Spring* (Carson 1962), the object of worry took on different forms; from a fear for pollution, greenhouse effects and loss of biodiversity, to a focus on the looming energy crisis, food insecurity and global warming (Edwards, 2010). Members of the counterculture and environmental organizations such as the Sierra Club in the US drew attention to these concerns, but environmental decline also became a point of debate among members of the American middle class and within political institutions (Hajer, 1995). In the early seventies, the policy-oriented but nevertheless ground-breaking *Limits to Growth* report was important in creating a new language and framing of environmental issues (Edwards, 2010; Hajer, 1995). This report put the risks of environmental decline and continuous economic growth on the global political agenda. But it also introduced cybernetic discourse and systems-thinking in political and cultural discussions about environmental crises. And most noteworthy for the genealogy that I sketch in this paper: it institutionalised the technocratic notion of the earth as a system and as a model that could be managed and improved.

The *Limits to Growth* report in 1972 clearly communicated the damaging effects of human activities on Earth and proposed limitations on economic growth. To share its radical message, the report used new computational technologies that allowed for large-scale modelling with the aim of predicting systems-change on a global scale (Edwards, 2010). These models were inspired by the work of Jay Forrester, who worked with a team called the Systems Dynamics Group at MIT on a dynamic computer model that aimed to represent the entirety of reality (Hajer, 1995, p. 81). With his team, Forrester developed iterations of his predictive model, called World 1, 2 and 3. The technique of climate modelling received critical scrutiny from scientists and activists, who critiqued the biases and limited variables within the models and their rudimentary state (Edwards, 2010, pp. 368–369). The *Limits to Growth* report was one of the first to use computer models, despite their major flaws, to share serious predictions and concerns about the exhaustion of finite resources, increasing pollution and world famine with a wider audience (Edwards, 2010, p. 368). The idea of a world model offered a clear concept to communicate bold and abstract ideas to wider audiences and create the feeling that the problems could be managed with the right techniques.

A discourse analysis shows that the *Limits to Growth* report was written in a novel language that borrowed heavily from cybernetics and its discussion of systems, complexity, feedback loops and more. The researchers describe their mission as a search for “the state of global equilibrium” (Meadows et al., 1972, p. 156). The earth is described as a “finite system” with positive and negative feedback loops affecting its state. The writers use the language of whole-systems to try to grapple with the complex issues they are dealing with and to study how different actions (input) would change the condition of the planet (output). They for example ask: “How will the world model behave if we include in it some policy to control growth deliberately? Will such a policy change generate a ‘better’ behavior mode?” (Meadows et al., 1972, p. 157). In terms of terminology and focus, the report reflected the ongoing changes in the field of ecology, as, for example, in the work of ecologists Tom and Eugene Odum who formulated holistic statements about the optimization of ecosystems (Kingsland, 2005, pp. 189–192). Their techno-optimistic vocabulary included cybernetic concepts such as equilibrium to discuss “the ‘essence’ of ecosystem dynamics” and discuss what “interventions would improve their operation, as though the systems were machines whose efficiency could be improved with a bit of tinkering” (Kingsland, 2005, p. 203). Thinking of the climate crisis and the planet in terms of equilibrium and stability discursively shaped environmental debates, for example promoting the idea that nature would “bounce back” into a healthy state, if humans would change their relation to and “use” of nature. The report thus introduced and helped institutionalize the language of whole-systems thinking into environmental discourse. With the wider acceptance of the world as a model, this also, unintentionally, presented an opening for techno-fixes to be introduced as the tools to maintain such stability. Aronczyk & Espinoza (2021, p. 133) therefore refer to the report as a “technology of legitimacy” that provided corporate PR actors with “information and influence methods” to frame sustainability as a theme. Because of its strong critique of economic growth, the report forced companies to demonstrate how their companies could function and thrive in this new reality (p. 134).

The discourse on environmentalism that the *Limits to Growth* report instigated, also had an influence on the counterculturalists. While the *Whole Earth Catalog* was no longer published regularly after 1971, Stewart Brand and his community still engaged with this new view on the environment. In their characteristically optimistic tone, they continued to celebrate the idea of ecosystem ecology in their next magazine project: *The CoEvolution Quarterly*, published between 1974 and 1984. Like the *Whole Earth Catalog*, the magazine published a range of tips, columns and book reviews that now also covered environmental topics such as vegetarianism, population growth and disaster prepping. Despite the attention for environmental decline, the tone of the magazine remained hopeful and continued to express a whole-systems worldview. In the introduction to the first edition, Brand wrote: “Ecology is whole system alright, but coevolution is whole system in TIME. The health of it is forward—systemic self-education which feeds on constant imperfection. We coevolving watchers and meddlers are not left out of it. Ecology maintains. Coevolution learns” (Brand, 1974). Co-evolution was thus presented as a particular “holistic” form of ecology that took environmental decline serious while still favouring progress and optimism, described in the language of whole-systems (Clarke, 2011).^2^

The wider adaptation of whole-systems thinking and its explicit connection to the climate crisis, mark a crucial step in the introduction and acceptance of technological solutions. With the Whole Earth community’s activities in the 1970s, it heralded a new era of techno-optimistic discourse that provided techno-fixes for a planet in peril, for example steering debates on environmentalism to a discussion of “mutual optimization” for both humans and the environment (Kirk, 2007, p. 165). This worldview became known as “ecopragmatism”, promoting “appropriate technologies” that would make co-evolution possible. Such solutions are still seen as more ecologically-aware forms of tech use that promote “responsible innovation”, yet the problem is that they also tend to look at environmental impact through a limited set of quantifiable aspects (Johnston, 2020).^3^

The analysis presented above shows how the “coupling” of nature and technology was strengthened by new discursive developments. While in the 1960s, the idea emerged that technology would be a partner of nature, the notion of co-evolution introduced in the 1970s, took this a step further. By speaking of the mutual optimization of humans and the environment, the future of both became strongly “coupled” together. Not only does this make technology and techno-fixes appear harmless and natural, whole-systems thinking now also created the idea that they evolve naturally. In addition, the political idea emerged that entrepreneurs and engineers, those with ecopragmatist visions, would best be suited to produce these solutions. I therefore agree with Johnston (2020) that it is vital to be critical of techno-fixes and their environmental rationale. Not only does ecopragmatism direct our attention to a limited set of climate solutions, it also changes how we discursively speak about the climate crisis. The introduction of techno-fixes would feed into the narrative that ecosystems (nature or society) could be solved by solutions that operate *outside* of that system (Johnston, 2020, p. 200). This is the process of “decoupling” technology from nature that became most clearly established in the 1990s.

## The 1990s: cyberutopianism and dreams of immateriality

In the period between the publication of the Club of Rome report and the commercialization of the internet that heralded an era of cyberutopianism, environmental debates in the US drastically changed in terms of tone and focus. Whereas the 1970s held on to the critical message of degrowth, the following decades saw a transition towards a more neoliberal approach to environmental issues. The changing economy and politics of the US in the 1980s, supported by President Ronald Reagan’s pro-growth agenda, led to new conceptualizations of the climate crisis and new understandings of “nature”. The Raegan administration heralded a time in which technological fixes were discussed with great optimism (Johnston, 2020, pp. 142–143). Key in this development was that nature had to be saved for its external values, its worth for humans and the economy, rather than for its intrinsic values (Dempsey, 2016, pp. 33–34). Such ideas emerged in political texts, but also, importantly, in corporate texts and PR communication, that purposely diffused the boundaries between climate speak and business speak, by promoting corporations as key agents in the climate crisis that set their own standards for environmental responsibility, shifting the attention from environmental issues to a discussion of how to define, manage and evaluate these issues (Aronczyk & Espinoza, 2021, pp. 180–181). Gómez-Baggethun and Naredo (2015) have noted three main shifts in this discourse between 1972 and 2012: the claim that economic growth is not bad but good for the environment; the shift in proposals from governmental climate regulations to market-based instruments; and an overall shift in language use from political to technocratic discourse. The third aspect is most clearly related to the prominence of Silicon Valley in the US in these decades.

Jessica Dempsey (2016) aptly describes the language shift to technocratic discourse. She argues that a problem such as biodiversity loss came to be understood as “a technical problem that can be fixed by finding a self‐sustaining equilibrium between the demands of consumers, national development interests, and the supply of global benefits stemming from biodiversity” (2016, p. 66). In an unrealistic fashion, the biosphere is asked to heal while serving everyone’s interests, including those of economic growth—a project that Dempsey critiques as unrealistically immaterial and placeless (2016, p. 49). This vision fits a broader North American narrative about growth and hegemonic power, displaying deeply patriarchal and colonial ideas about who “owns” nature. Scholars, including ecofeminists and indigenous scholars, have critiqued this rationale about the mastery of nature (Haraway, 2016; Merchant, 1980; Plumwood, 1993; Shiva, 1997). Such “pro-growth” environmentalism is described by Hajer (1995) as ecological modernization, while Jessica Dempsey (2016) speaks of “enterprising nature”, and Kathleen McAfee (1999) of “green developmentalism”. This optimistic worldview that is deeply steeped in neoliberalism prioritizes economic growth, values nature for its instrumental and economic use, and believes in technological innovations that will save humanity. As McAfee (1999, p. 133) argues, this narrative imagines that human’s interests all align, and that environmental issues can be solved without confronting current economic models. In this narrative, it makes sense to forward new technologies as “god tricks”, creating an objective and distanced perspective on environmental issues. I therefore argue that this particular narrative overlaps with whole-systems thinking, imagining techno-fixes as tricks that are decoupled from the (eco)system they aim to improve.

In the 1990s, the countercultural ideals of the 1960s were transformed into a neoliberal ideology that had much in common with the military-industrial complex whose politics and bureaucracy the movement had long opposed. Turner (2006) explains that despite some clear political differences, Republicans in the 1990s were, like the new communalists in the 1960s, highly interested in the possibilities technologies offered to individuals, businesses and alternative forms of governance. This development was reinforced by the popularization of the Internet, which created the widespread belief that technology could save or upgrade humans and the environment. When the Internet became widely accessible and gained a more central role in popular culture in the 1990s, a new sentiment emerged towards the internet as a virtual place and tool for human freedom.

Barbrook and Cameron’s text (1996) on the Californian Ideology captured this sentiment as a mix of a hippie and entrepreneurial spirit. This spirit is embodied by figures such as Stewart Brand, but also Kevin Kelly, editor of *CoEvolution Quarterly* and later *Wired* magazine, and writers such as Esther Dyson and John Perry Barlow (Turner, 2006). Their projects and writings illustrate the rise of digital utopianism, that intensified the use of whole-systems thinking within the ecomodernist dream of decoupling. Barlow perhaps best represents the techno-optimism of the 1990s. As author of the famous text “Declaration of the Independence of Cyberspace” (Barlow 1996), he presented cyberspace as an immaterial world operating completely separated from the “real” world. Barlow’s text also offers a political vision, as his cyberlibertarian and transhumanist dreams about a world without governmental interference are central to his cyber-utopian dream. It explicitly “decouples” cyberspace from the constraints of the material world and creates a condition of virtuality in which information and materiality are fiercely separated, as Hayles (1999, p. 20) so critically discusses. This narrative allows Barlow to not deal with the physical manifestations and effects of the internet, erasing both its history and its materiality. This erasure is a key element in discussions of the history and materiality of the internet, illustrated by the myth of the internet as ephemeral “cloud” (Ensmenger, 2018; Hu, 2015).

In my view, the cyberutopian ideology not only obscures the materiality of the internet, it also produces a particular understanding of the environment in computational terms. I argue that this is a datafied vision on the planet; a view that is represented by what writer Kevin Kelly (1998) referred to as the “computational metaphor”. He describes the emergence of a “collective journey toward the belief that the universe is a computer” (n.p.). In his view, seeing everything as a computer, is a transformation in language that would help to describe all kinds of phenomena. Of course, he writes, he knows that the universe is not actually a computer, “only that it may act as if it is one. But once the metaphor of computation infiltrates physics and biology deeply, there is no difference between those two statements. It’s the metaphor that wins” (Kelly, 1998, n.p.). The metaphor of a computational planet has turned out to be a very powerful one, with real material consequences that Kelly perhaps did not consider.

More and more, computer technology has become the lens through which the world is understood. Cyberutopian discourse offers a simplistic appropriation of cybernetic ideas, as the “rich discourse of cybernetics and information theory was flattened in the utopian information narrative” (Kline, 2015, p. 7). The techno-optimistic if not utopian discourse ignores that cybernetics, which had been fading as a research field since the 1970s, had in fact paid attention to the energy needed, the costs and the environmental efforts required to sustain complex (eco)systems. Already in the 1980s, some people became aware that the computer industry was affecting the environment and was not as clean compared to older industries (Parikka, 2015). But within Silicon Valley culture, these environmental considerations remained largely undiscussed until the early 2010s. The imagined immateriality of technology and the world-as-computer-idea that became dominant in the 1990s allowed Silicon Valley to ignore its own impact on the climate. This decoupling of technology and materiality in fact helped to position the industry as environmentally beneficial and as a key actor in the climate crisis. The language of whole-systems enabled companies to uphold this myth, by separating the physicality of natural ecosystems from the virtuality of the internet.

## From past to present: technofixes as part of contemporary green capitalism

I have demonstrated how techno-optimism throughout key moments in the 1960s, 1970s and 1990s has emerged as an answer to environmental dilemmas, creating the idea that technologies can help save and manage nature. This optimism emerged at the intersection of Silicon Valley’s tech discourse and socio-political environmental discourse. Based on my discourse analysis and literature review, I argue that to understand the development of techno-optimistic discourse, it is crucial to zoom in on the use and development of whole-systems thinking. This language has emerged in popular tech discourse from the 1960s onwards and has brought environmental and technological questions together.

As I have shown, whole-systems language and its ecomodernist ideology is embedded in a longer history of techno-optimism. The Whole Earth community of the 1960s brought this techno-optimism to a wider community, promoting libertarian environmentalism through a language of systems-thinking inspired by cybernetics and ecology. With the groundbreaking *Limits to Growth* report, the language of systems-thinking entered environmental discourse, showing how economic and social changes were necessary to prevent environmental decline. In the 1990s, unbridled techno-optimism further naturalized the idea that “immaterial” technologies could bring a more democratic, creative, and environmentally friendly world about. While the history of Silicon Valley’s environmental ideology cannot be reduced to a small number of actors and events, the partial genealogy of whole-systems thinking that I have sketched out in this article does show that today’s ideology has a history that deserves further exploration. By thinking of the world at large, but also natural ecosystems, societies, communities and organizations in terms of systems, techno-optimistic discourse has created the idea that technology can operate as a partner of nature by emphasizing its naturalness and immateriality, while at the same time the natural environment is imagined as a simplified system that would, metaphorically, work like a computer. Returning to Marc Andreessen’s “The Techno-Optimist Manifesto” (2023), it becomes apparent how this narrative reappears in his vision of nature. His vague and at times contradictory statements, seeing the economy as a system, and energy in terms of abundance, allow him to create a simple and positive story about innovation and the climate crisis.

Interestingly, Marc Andreessen himself is a part of the history I have described here, as co-founder of the popular web browsers Mosaic and Netscape in the early 1990s. Later, he started the venture capitalist firm Andreessen Horowitz (also known as A16z), that invested in companies such as Facebook, Airbnb and Lyft (Friend, 2015). Andreessen is therefore representative of what I refer to as the region and cultural phenomenon Silicon Valley. In recent years, Silicon Valley has expanded into a “syndicate” that is not restricted by the geographical boundaries of the San Francisco region: it represents a conglomeration of Big Tech businesses, infrastructural agents and individuals that have or have had ties to these companies. Andreessen’s venture capitalist firm is at the heart of this economic system. Named after the technical infrastructures that these companies control and manage, their platforms, this economic model has come to be known as “platform capitalism” (Srnicek, 2016; Van Dijck et al., 2018). The environmentalism of Silicon Valley is inherently tied to its economic interests, which motivates companies to position technological innovations and their own products and services as solutions to the climate crisis. Since the 2010s, Big Tech companies have developed an agenda of “green capitalism”, promoting their companies not only as agents that bring their users connectivity and efficiency, but even sustainability (Buller, 2022; Goldstein, 2018; Hogan, 2018). Through carefully designed PR campaigns, Silicon Valley has made an enormous effort to “rebrand” itself as sustainable industry (Aronczyk & Espinoza, 2021; Buller, 2022; Goldstein, 2018; Morrison, 2020). Again, whole-systems thinking plays a central role in this process of rebranding.

The environmental dimension of platform capitalism and Silicon Valley’s techno-optimistic discourses lead me to reconceptualize the ideological underpinnings of Silicon Valley, described as the “Californian Ideology” by Barbrook and Cameron (1996). I therefore want to return to the ideology of ecomodernism and its belief that human progress can be “decoupled” from environmental decline. In my view, this is a deeply optimistic dream that reveals a strong belief in the potential of and the need for unbridled technological innovation. As Barbrook and Cameron (1996) already concluded in relation to the Californian Ideology, the ecomodernist ideology of Silicon Valley is full of contradictions. While the companies and individuals present themselves as environmentalists that want to protect natural ecosystems they say they are part of, they propose pro-growth futures that further damage these same ecosystems and imagine their techno-fixes as external solutions. When Andreessen or the authors of “An Ecomodernist Manifesto” speak of separating the techno-capital machine or peak human impact from nature, they formulate smart rhetorical statements that only work on paper. I therefore agree with Anne Pasek (2019, p. 9) that we need to scrutinize the aspirations of tech companies and their “strategies of withdrawal and erasure”. As Mél Hogan (2018, p. 648) argues, the concept of the global ecosystem can help us to critically assess the environmental impact of Big Tech, but it is also used to rationalize “the domination of machine logics over nature” and present Big Tech as a self-contained system, best employed to manage and utilize nature. I argue that we should understand this in terms of “uncoupling” and “decoupling” nature from culture: discursive strategies that have come to underlie whole-systems thinking in contemporary techno-optimistic discourse.

Ecomodernism is a Western, modernist worldview that celebrates human freedom, including freedom from nature, above everything else (Crist, 2016). In its trust in technological innovations, it echoes the optimism and search for harmony of the 1960s, the dream of co-evolution of the 1970s and the managerial perspective on nature and internet utopianism of the 1990s. And while it acknowledges climate change as an existential crisis, it turns it into an opportunity for green growth paired with increased well-being. In ecomodernist writings, it remains unclear why absolute decoupling is desirable, let alone how it would be realized, nor who would actually profit from it. It may come as no surprise that Whole Earth editor Stewart Brand is co-signer of “An Ecomodernist Manifesto”; he is also author of the book *Whole Earth Discipline: An Ecopragmatist Manifesto* (Brand 2009). Stewart Brand embodies the ecomodernist mindset. He has taken a role as futurist, sharing his optimistic vision on tech-fixes for the climate, for example through geo-engineering and terraforming the earth, stubbornly overlooking its risks and shortcomings (Johnston, 2020, pp. 213–215). While I agree with Kirk (2007) that Brand has been an important figure for American environmentalism, I am more critical of the pragmatic and capitalistic underpinnings of his worldview.

Returning to the other examples of tech-on-climate discourse that I started this paper with, the Bezos Earth Fund and Microsoft, I can now situate them within the same ecomodernist mindset that privileges techno-optimism and whole-systems thinking. The most prominent agents in the American landscape of Big Tech have adopted a positive and comforting story about co-evolution, systemic change and progress in order to decouple their businesses from its large environmental impact. Both the “Systems Change Lab” (Bezos Earth Fund, 2023) of the Bezos Earth Fund and the “Planetary Computer” (2020) by Microsoft make clear that Silicon Valley discusses the climate crisis as a crisis for which humanity lacks the quantafiable information and technological tools to “know” and thus save the planet and its complex ecosystems. Not suffering from doubt or humility, they offer large solutions and propose systemic changes without however taking the necessary steps to achieve such radical change. This is not surprising, as the logic of green (platform) capitalism shows that they might change tactics, but not their growth-oriented business model (Goldstein, 2018).

## Conclusion

By exploring three seminal moments in the environmental history of Silicon Valley, I have demonstrated how today’s tech-on-climate discourse has in many ways its roots in the Californian tech culture from the 1960s onwards. Under a veneer of ecosystem-marketing we find, I argue, an ideology of ecomodernism that imagines a unified, superior humanity reigning over the natural world; a world imagined as a closed system that can be quantified and managed. This ideology is wrapped in a whole-systems language that promotes technological solutions as pragmatic and realistic, setting aside other perspectives as utopian or naïve. I have called this “decoupling”: an ecomodernist term that operates as a discursive strategy to separate nature from culture. Through strategic forms of coupling and decoupling, Silicon Valley actors direct our attention to positive connections they want to highlight, while erasing others. Based on my analysis of three key moments in the history of this discourse, I formulate three points of critique.

Firstly, I critique the notion of the datafied planet. I have shown that new communalists in the 1960s were inspired by cybernetic thinking, because it provided them with a harmonious and reassuring perspective on the world. In small communities and with the right technologies, a peaceful world could be created. Technologies could be of help here, because they came to be understood as part of nature, and not in opposition to nature. While the Whole Earth community talked about small-scale technologies, large companies like Microsoft now use similar arguments to sell technical tools such as the Planetary Computer, which according to Microsoft allows us to collect and analyse the data necessary to “understand” our environment in decline. But this naturalized understanding of technology makes it hard to critique the use of technologies. It has also created the illusion that nature and information about nature are the same thing, and that the world, understood as a “program earth” can thus be known and managed with information (Gabrys, 2016). This narrative gives a false sense of security about how information can help us know and understand planetary conditions. This “honeymoon objectivity”, as Sun-ha Hong (2020) calls it, has gradually changed and is still changing our understanding of what knowledge is, which has consequences for the discussion on the solutions for climate change. Today’s environmental discourse in Silicon Valley has successfully reframed the problem, drawing our attention to how problems can be made visible, measurable and how they can be managed with certain solutions, that then can be measured and evaluated on their usefulness. Redefining the problem is one side of the coin, redefining the solutions is the other.

Secondly, I critique the appropriation and simplistic use of terms such as “ecosystem” in whole-systems discourse, which hides the modernist and colonial perspective on humans as rulers of nature. In the 1970s debates on degrowth, systems thinking became a tool to understand the world and the consequences of large-scale industrial activities. But despite the emergence of systems science and the use of models in climate policies, whole-systems language uses a simplified understanding of biological processes, portraying climate change as an easily solvable problem. With their misleading focus on the restoration of balance within a growth economy, today’s manifestation of this techno-optimistic discourse does not actually deliver a situated perspective on the interdependencies between humans and non-humans. Instead, it offers a “cybernetic-ecosystemic dream” that sees the world as input for the production and consumption that Silicon Valley sustains (Hogan, 2018). By doing so, it maintains the disembodied perspective that arose in the 1990s as part of cyberutopianism.

Thirdly, I critique Big Tech’s teleological narrative, that renders alternative future paths or different climate solutions irrelevant or invisible. Ecomodernism provides a positive tale of how tech companies can engineer the world out of the climate crisis, but not without sketching the risk of an apocalyptic future that might happen if their proposals are not followed up (Goldstein, 2018; Zylinska, 2018). Their narrative is therefore both imperialistic and masculinist, embodied by figures such as Marc Andreessen, Elon Musk and Jeff Bezos (Little & Winch, 2021). It conceptualizes Silicon Valley as a leading force that knows best how to “fix” the climate crisis and realize a “better” future, in which companies can maintain and expand their current economic activities. In a true ecomodernist sense, actors including Andreessen, but also OpenAI CEO Sam Altman (Dastin, 2024), claim that we need an abundance of renewable energy to power the ever-expanding infrastructures of Big Tech and the ever-increasing hunger for energy and materials that keep the services, including the recent surge of AI services, going. The call from the 1970s to look for moderation has been replaced by a call for abundance that does not take the environmental limitations of renewable energies into account, but is nevertheless portrayed as beneficial to the environment. In another recent moment from this environmental discourse, Apple (Producer) (2023) launched a video with a bold environmental message, in which the personification of Mother Nature congratulates the company on its climate progress.

With the growing cultural production of “tech-on-climate discourse”, I advocate that in future research we should continue to draw attention to Silicon Valley’s history as a place and birth ground of a specific environmental ideology. Despite increasing attention for degrowth and postgrowth narratives in society as a whole, the discursive power of Big Tech platforms continues to promote an ecomodernist ideology that puts emphasis on a future of green capitalism while maintaining further growth. As the ecomodernist dream of decoupling technology from environmental impact keeps re-inventing itself, it requires us to continuously *recouple* the utopian narratives of Big Tech with their material consequences.^4^

## Notes

1 Don Ihde (1990) has used the term “single system utopianism” to refer to something similar: the obsession to simplistically conceptualize processes as systems, including the “whole Earth”.

2 Whereas designer Buckminster Fuller had been the central inspiration for the *Whole Earth Catalog*, anthropologist and cybernetician Gregory Bateson and his “cybernetic epistemology” became the inspiration for *The CoEvolution Quarterly* (Clarke, 2011).

3 Despite the good intentions of his ecopragmatism, Brand pushed the environmental agenda beyond small-scale solutions, by simultaneously promoting proposals for space colonies, a controversial plan that illustrates Brand’s interest in adventurous projects and his affinity with libertarian politics (Kirk, 2007, pp. 170–175).

4 I am inspired here by the call by Gómez-Baggethun & Naredo (2015, p. 393) who write: “Such a project may only be accomplished following an epistemological break in economic thinking that recouples the economy with planetary boundaries by acknowledging limits to the substitution of natural resources and the unaccounted social and ecological costs of growth”.
